# Comparative Finite Element Analysis of Denosumab and Bazedoxifene on Pedicle Screw Stability in Osteoporotic Spines

**DOI:** 10.1002/jsp2.70147

**Published:** 2025-12-09

**Authors:** Tomoyuki Asada, Soji Tani, Tomoko Towatari, Mahoko Ishikawa, Philip Varnadore, Yoshifumi Kudo, Peter G. Passias, Benjamin A. Alman, Koji Ishikawa

**Affiliations:** ^1^ Hospital for Special Surgery New York New York USA; ^2^ Department of Orthopedic Surgery Showa Medical University Tokyo Japan; ^3^ Department of Orthopaedic Surgery Duke University Durham North Carolina USA

**Keywords:** bazedoxifene, Bone quality optimization, denosumab, finite element analysis, osteoporosis, pedicle screw fixation, spinal instrumentation

## Abstract

**Introduction:**

Pedicle screw fixation in osteoporotic spines remains challenging. Bazedoxifene (BZA) and denosumab (Dmab) are widely used agents for osteoporosis, but their comparative effects on spinal instrumentation are not well understood. This study aimed to evaluate the effects of BZA and Dmab on biomechanical parameters of spinal instrumentation using finite element analysis (FEA).

**Methods:**

In this prospective, open‐label study, postmenopausal women with primary osteoporosis were assigned to receive either BZA (daily oral, 20 mg) or Dmab (subcutaneous, 60 mg every 6 months) for 12 months. FEA models of the L4 vertebra were generated from CT scans using a calibration phantom (Mindways, Austin, TX, USA). Vertebral compression force was evaluated to represent overall vertebral strength. Pedicle screw fixation strength was assessed under axial (pullout strength) and non‐axial directional forces (cranial, caudal, lateral, medial). Inverse probability of treatment weighting (IPTW) and multivariable regression were used to balance baseline differences and compare biomechanical outcomes between groups.

**Results:**

Thirty patients were enrolled (15 per group); the final analysis included 12 in the BZA group and 13 in the Dmab group. Compared to BZA, Dmab significantly improved compression strength (adjusted mean difference: 8.1% [95% CI, 0.9–15.3], *p* = 0.030) and pullout strength (15.8% [95% CI, 6.2–25.4], *p* = 0.003). Directional FEA revealed greater resistance to cranial (17.4% [95% CI, 4.9–30.0], *p* = 0.009) and lateral (10.8% [95% CI, 0.9–20.8], *p* = 0.035) loading with Dmab, while no significant difference was observed in caudal‐ and medial‐directed force.

**Conclusion:**

Finite element modeling suggested that Dmab enhanced pedicle screw fixation more effectively than BZA, particularly against axial and cranial/lateral‐directed forces. These biomechanical differences underscore the potential advantage of Dmab in preoperative osteoporosis management to improve pedicle screw stability.

## Graphical abstract

The study aims to compare the effects of denosumab and bazedoxifene on spinal instrumentation surgery. CT scans obtained before and after treatment were used to generate 3D vertebral models for in silico biomechanical analysis, and mechanical loading directions—including compression and multidirectional screw forces—were applied to assess implant stability.

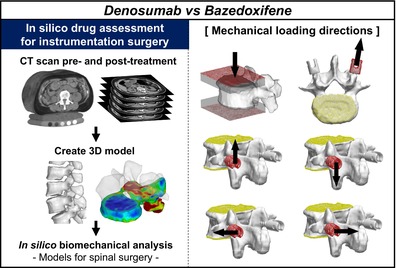

## 1 Introduction

Osteoporosis significantly impacts outcomes in spinal surgery, particularly when instrumentation such as pedicle screws is used, often leading to fixation failure and increased reoperation [1]. Optimizing preoperative bone quality through pharmacologic intervention is essential to mitigate these risks [2, 3]. Antiresorptive medications including bisphosphonates, selective estrogen receptor modulators (SERMs), and denosumab (Dmab) are widely used for primary postmenopausal osteoporosis, and are expected to play a role in preoperative bone optimization in spinal fusion surgery [4]. Although bisphosphonates are first‐line therapies for primary osteoporosis, their side effects and inconvenient administration often limit adherence, prompting the use of alternatives such as SERMs and Dmab [5, 6]. These medications effectively improve vertebral strength and reduce the risk of osteoporotic vertebral fractures [7, 8, 9, 10].

Bazedoxifene (BZA), a third‐generation SERM, acts as a highly selective estrogen receptor agonist in bone tissue, where it inhibits osteoclast activity and suppresses bone resorption, thereby preserving bone mass [11]. Dmab, a monoclonal antibody with high affinity for RANKL, inhibits bone resorption by blocking the interaction between RANKL and its receptor RANK, thereby preventing osteoclast formation and activity.

These medications are clinically beneficial, but direct comparative data on their effects in spinal instrumentation surgery are limited. Using finite element analysis (FEA), we demonstrated that Dmab significantly improves pedicle screw pullout strength (POS) following 2‐year treatment [12]. However, no prior studies have directly compared the biomechanical effects of different antiresorptive agents. Therefore, this study aimed to investigate the effects of BZA and Dmab on vertebral and pedicle screw strength in osteoporotic patients, to better understand treatment selection in spinal surgery.

## 2 Materials and Methods

### 2.1 Study Design and Patients Selection

This was a prospective, non‐randomized, open‐label study conducted between February 2018 and January 2022, involving patients with primary postmenopausal osteoporosis. The study was approved by the ethics committee of Yamanashi Red Cross Hospital and conducted in accordance with the principles of the Declaration of Helsinki. Written informed consent was obtained from all participants prior to any study‐related procedures.

Inclusion criteria were: (1) age ≥ 60 years, (2) diagnosis of primary osteoporosis, and (3) no prior history of osteoporosis treatment. Exclusion criteria included: (1) suspected secondary osteoporosis based on baseline assessments, (2) a medical history of musculoskeletal disorders such as rheumatic disease or Parkinson's disease, (3) use of medications that may affect bone metabolism, such as corticosteroids, (4) poorly controlled thyroid disease, (5) active malignancy under treatment, or (6) history of surgery within the past 6 months. We selected patients ≥ 60 years to align with the typical surgical population in which osteoporosis is highly prevalent [13]. Secondary osteoporosis was screened via structured interview basis, using a standardized FRAX‐based questionnaire.

Power calculations were based on vertebral compressive strength measured by QCT‐based FEA, the same biomechanical model evaluated in the reference study [14]. In that study, Dmab significantly improved whole vertebral body strength, with a reported effect size of 1.51 (95% CI, 1.20–1.88) referred to a ratio of means for vertebral compressive strength between Dmab and the vehicle group. Using the log‐transformed ratio and the reported variance on the log scale from that report, a two‐sample *t* test (*α* = 0.05, 80% power) indicated a requirement of nine participants per group. Accounting for an expected 20% dropout rate yielded a target of approximately 12 per group. Because the present study compared Dmab with an active comparator (BZA) rather than vehicle [15], we pragmatically increased the target to 15 participants per group to preserve power and improve the precision of estimates [16, 17]. Other endpoints were considered exploratory, and no separate sample‐size calculations were performed due to the limited availability of prior data.

Patients were allocated to either the BZA or Dmab group at the time of diagnosis based on their preference after treating physicians provided a neutral explanation of the two treatment options covering their mechanisms of action, administration schedules, and potential side effects. Treating physicians explicitly refrained from recommending one option over the other. Dmab (60 mg) was administered subcutaneously every 6 months for 12 months (two injections per patient) by trained clinical staff, whereas BZA (20 mg) was taken orally once daily by patients for 12 months. All patients received daily supplementation with eldecalcitol 0.75 μg/day and/or calcium l‐aspartate hydrate 400–800 mg/day to prevent Dmab‐induced hypocalcemia and to avoid potential bias from differences in vitamin D and calcium status that could affect bone metabolism and study outcomes [18, 19].

### 2.2 Data Collection

Patient baseline demographic included, age (year), body mass index (BMI, kg/m^2^), history of fragility fracture, family history of femoral or vertebral fracture, and baseline VAS back score. We evaluated serum levels of albumin (g/dL), calcium (Ca, mg/dL), phosphorus (IP, mg/dL), estimated glomerular filtration rate (eGFR, mL/min/1.73 m^2^), alkaline phosphatase (ALP, U/L), and intact parathyroid hormone (iPTH, pg/mL) before treatment. Serum bone turnover markers (BTMs) included tartrate‐resistant acid phosphatase type 5b (TRACP‐5b; reference range in women, 120–420 mU/dL; estimated using Osteolink TRACP‐5b test kit; DS Pharma Biomedical Co Ltd., Osaka, Japan) and total *N*‐terminal propeptide of type 1 procollagen (total P1NP; reference range in postmenopausal women, 26.4–98.2 μg/L; estimated using a total P1NP assay on an Elecsys automated analyzer; Roche Diagnostics, Switzerland), which were assessed at baseline and 12 months after treatment initiation.

Dual‐energy X‐ray absorptiometry (DXA) of the lumbar spine, femoral neck, and total hip was performed at baseline, and 12 months. Areal bone mineral density (BMD) of the lumbar spine (L1–4) (spine BMD), femoral neck (femoral neck BMD), and total hip (total femur BMD) was measured using DXA (Hologic QDR series, Hologic Waltham, MA, USA). All DXA measurements were analyzed by a radiologist at a central site.

### 2.3 FEA

Three‐dimensional finite element models of the L4 vertebra were generated from the CT data using Mechanical Finder software (version12.0 extended edition; Research Center of Computational Mechanics, Tokyo, Japan). CT data were acquired with Revolution EVO ES (GE healthcare) using predefined scanning conditions (x‐ray energy, 120 kV; x‐ray current, SD20; rotation speed, 0.8 s/rot; beam pitch, 0.984; slice thickness, 2 mm; reconstruction intervals, 2 mm). For QCT scanning, a phantom (Mindways, Austin, TX, USA) was placed underneath the patients for BMD calibration, thereby ensuring measurement quality throughout the study. The FEM modeling and analysis methods followed protocols established in previous studies [3, 12, 20, 21, 22]. The models were meshed with 2‐mm tetrahedral solid elements and 2‐mm triangular plates [23]. Mesh convergence analysis was performed in our recent study and confirmed the consistency of volumetric BMD values across mesh sizes ranging from 1.2 to 3.0 mm [3]. In addition, a practical mesh‐size check for biomechanical parameters using four representative sizes (1.2, 2.0, 2.5, and 3.0 mm) showed < 14% variation in compression force (Figure S1a) and < 13% in POS (Figure S1b). These results support the adequacy of a 2.0‐mm element size for the primary mechanical parameters in this dataset.

For the plates, a Young's modulus of 10 GPa and a thickness of 0.4 mm were assigned. Bone heterogeneity was incorporated by calculating the BMD of each element from its Hounsfield unit value. Element‐specific Young's modulus and yield stress were obtained from Keyak et al., with Poisson's ratio set to 0.4 [24]. Specifically, the constitutive equations used were

$$\text{Elastic modulus}:E=\left\{\begin{array}{c} 0.001\;\left(\rho =0\right)\\ 33900\rho^{2.20}\left(0<\rho \leq 0.27\right)\\ 5307\rho +469\left(0.28<\rho \leq 0.6\right)\\ 10200\rho^{2.01}\left(0.6\leq \rho \right) \end{array}\right\}$$

$$\text{Yield stress}:\sigma =\left\{\begin{array}{c} 1.0*10^{20}\;\left(\rho \leq 0.2\right)\\ 137\rho^{1.80}\left(0.2<\rho \leq 0.317\right)\\ 114\rho^{1.72}\left(0.317<\rho \right) \end{array}\right\}$$

These material properties were group‐consistent, with patient‐specific variation arising solely from the individual BMD distribution.

**FIGURE 1:**
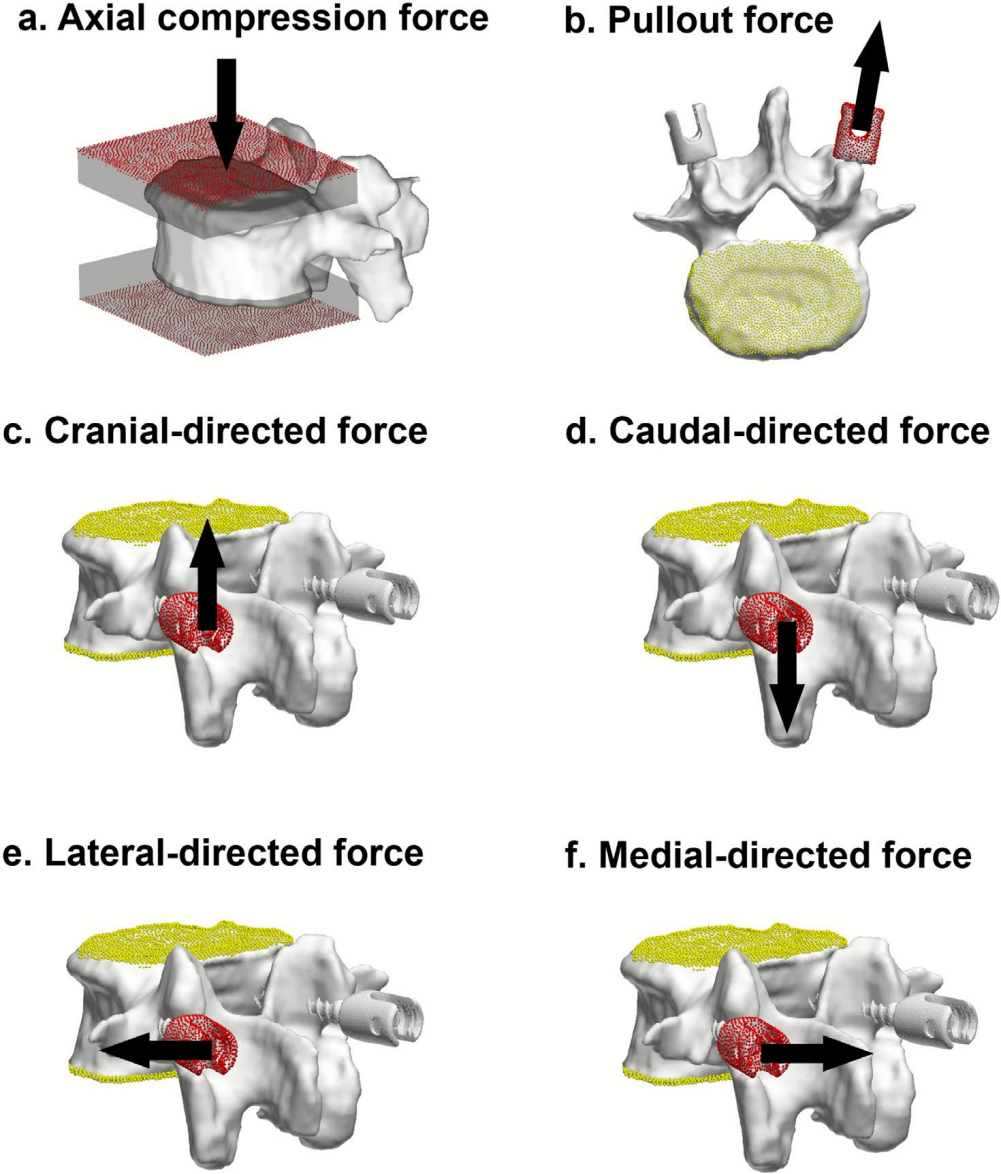
Loading directions for compression and pedicle screw testing. (a) Axial compression force, (b) pullout strength (POS), (c) cranial‐directed force, (d) caudal‐directed force, (e) lateral‐directed force, and (f) medial‐directed force. Compression was applied to the cranial PMMA cap; pullout and multidirectional loads were applied to the screw head.

A compressive displacement was applied incrementally (0.01 mm steps) to a poly(methyl‐methacrylate) (PMMA) cement cap at the cranial surface of the vertebrae. The caudal PMMA surface was fully fixed. The predicted vertebral compression strength was defined as the peak force on the force–displacement curve, immediately preceding a sustained post‐peak decline in load‐bearing capacity (Figures 1a and S2) [3, 12, 22, 23].

PS fixation was assessed using the same implant (ZODIAC‐Spinal Fixation System, Alphatec) across all models. Screws measured 40 mm in length and 7.5 mm in diameter. Screw models were reconstructed from micro‐CT and meshed with 1.0‐mm tetrahedral elements. Material properties of titanium alloy were applied (Young's modulus of 110 GPa, yield stress of 900 MPa, and Poisson's ratio of 0.28). Screws were inserted along the pedicle axis and parallel to the endplate using Weinstein's technique [25], while vertebral endplates were fully constrained. Material properties were summarized in Table S1.

To assess POS, tensile loading was applied at 20 Newton (N) per increment to the screw head along the screw axis. POS was defined as the peak load immediately before a marked change in the slope of the load–displacement curve [3, 12, 22] (Figure 1b).

To evaluate the fixation strength against multidirectional loading, loads of up to 200 N were applied at an incremental rate of 20 N per step cranial/caudal parallel to the vertebral axis, medial/lateral orthogonal to the vertebral axis (Figure 1c–f). Screw fixation strength was defined as the load at the inflection point on the load–displacement curve immediately before an abrupt increase in displacement [21]. The inflection point was identified manually by an investigator blinded to group allocation using a predefined visual criterion, and the markings were verified by another author.

Treatment effects of Dmab and BZA were incorporated into the FE models by updating vertebral geometry and element‐wise material properties based on follow‐up CT‐derived changes in volumetric BMD and morphology, although no substantial changes in vertebral morphology were expected.

### 2.4 Statistical Analysis

All statistical analyses were performed using R (version 4.4.0; R Core Team 2024, Vienna, Austria). Given the sample size, data distribution was assessed using the Shapiro–Wilk test. Continuous variables were reported as mean ± standard deviation (SD) or median with interquartile range (IQR), as appropriate. Categorical variables were summarized as frequency and percentage (%).

Comparisons between the two treatment groups were conducted using Welch's *t* test or the Mann–Whitney *U* test, depending on the data distribution. Differences between groups were described by standardized mean differences (SMD). For longitudinal within‐group comparisons, paired t‐tests or Wilcoxon signed‐rank tests were applied. Correlation analysis was conducted with Pearson or Spearman analysis based on the distribution of variables.

For FEA comparisons, inverse probability of treatment weighting (IPTW) based on the average treatment effect (ATE) was employed to mitigate potential bias from baseline differences, given the non‐randomized, open‐label study design. Propensity scores were calculated by a logistic regression model including age, BMI, spine DXA *T* score, pretreatment albumin level, and history of fragility fracture. The primary analysis used unstabilized IPTW with a light upper winsorization at the 95th percentile to mitigate extreme weights. Covariate balance was assessed using SMDs before and after weighting. Treatment effects were estimated using weighted multivariable regression analysis with design‐based Huber–White (sandwich) robust standard errors (SEs), adjusting for age, BMI, spine DXA *T* score, pretreatment albumin level, and history of fragility fracture. Sensitivity analyses included (i) unweighted analysis of covariance (ANCOVA) with the same covariates without IPTW and (ii) stabilized IPTW with the same covariates and robust SE. The results of the multivariable regression were described with estimate (adjusted mean difference in group variable, *β*) ± SE, 95% confidence intervals (95% CIs) and *p* value.

All statistical tests were two‐tailed, and statistically significant was set as *p* < 0.05.

## 3 Results

### 3.1 Baseline Patient Characteristics

A total of 30 postmenopausal patients without prior osteoporosis treatment were enrolled, with 15 patients each selecting either BZA or Dmab. Five patients dropped out due to moving (*n* = 1) or loss of motivation (*n* = 4). Consequently, 12 patients in the BZA group and 13 patients in the Dmab group completed the 12 months follow‐up and were included in the final analysis (Figure S3). There were no significant baseline differences between the groups in age (BZA: 70.2 ± 9.6 vs. Dmab: 68.8 ± 4.6 years), BMI, albumin, serum calcium, TRACP‐5b, or total P1NP. Although not statistically significant, the Dmab group tended to have a numerically higher proportion of patients with a history of fragility fracture (BZA: 25.0% vs. Dmab: 38.5%), higher baseline total P1NP (BZA: 56.0 ± 16.4 vs. Dmab: 70.8 ± 33.4), and lower spine DXA *T* scores (BZA: −1.6 ± 1.0 vs. Dmab: −2.1 ± 0.9) (Table 1).

**TABLE 1:** Demographic data.

	BZA	Dmab	*p*	SMD
Age, years (mean, SD)	70.2 (9.6)	68.8 (4.6)	0.66	0.18
BMI, kg/m^2^ (mean, SD)	22.7 (3.4)	24.0 (3.8)	0.37	0.37
History of fragility fracture, *n* (%)	3 (25.0)	5 (38.5)	0.67	0.29
Family history, *n* (%)				
Femur	0 (0.0)	2 (15.4)	0.48	0.60
Lumbar spine	1 (8.3)	0 (0.0)	0.48	0.43
VAS back, mean (SD)	2.3 (2.2)	1.5 (1.9)	0.38	0.37
Alb (g/dL), mean (SD)	4.3 (0.3)	4.4 (0.2)	0.31	0.41
Ca (mg/dL), mean (SD)	9.3 (0.3)	9.4 (0.2)	0.37	0.36
IP (mg/dL), mean (SD)	3.6 (0.5)	3.6 (0.4)	0.75	0.13
eGFR (mL/min/1.73 m^2^), mean (SD)	73.5 (12.5)	75.3 (18.0)	0.78	0.12
ALP (U/L), median [IQR]	279.5 [207.8, 298.2]	269.0 [228.0, 308.0]	0.62	0.11
intact‐PTH (pg/mL), median [IQR]	40.0 [36.0, 46.5]	35.0 [29.0, 52.0]	0.79	0.27
TRACP‐5b (mU/dL), median [IQR]	450.0 [386.2, 505.0]	474.0 [390.0, 629.0]	0.55	0.48
Total P1NP (μg/L), mean (SD)	56.0 (16.4)	70.8 (33.4)	0.18	0.56
Homocysteine (nmol/mL), median [IQR]	8.7 [7.9, 11.1]	8.6 [7.7, 12.5]	0.69	0.35
DXA				
Spine BMD, mean (SD)	0.8 (0.1)	0.8 (0.1)	0.19	0.55
Spine *T* score, mean (SD)	−1.6 (1.0)	−2.1 (0.9)	0.18	0.56
Femoral neck BMD, mean (SD)	0.6 (0.1)	0.6 (0.1)	0.39	0.35
Femoral neck *T* score, mean (SD)	−2.2 (0.7)	−2.5 (0.7)	0.42	0.33
Femoral total BMD, mean (SD)	0.7 (0.1)	0.7 (0.1)	0.93	0.03
Femoral total *T* score mean, (SD)	−1.8 (0.9)	−1.8 (0.7)	0.94	0.03

*Note:* Continuous variables with non‐normal distributions were presented with median [IQR].

Abbreviations: BMD, bone mineral density; BMI, body mass index; BZA, bazedoxifene; Dmab, denosumab; DXA, dual‐energy X‐ray absorptiometry; IQR, interquartile range; SD, standard deviation; SMD, standardized mean difference.

### 3.2 Changes in Bone Turnover Markers and DXA After 1 Year

**FIGURE 2:**
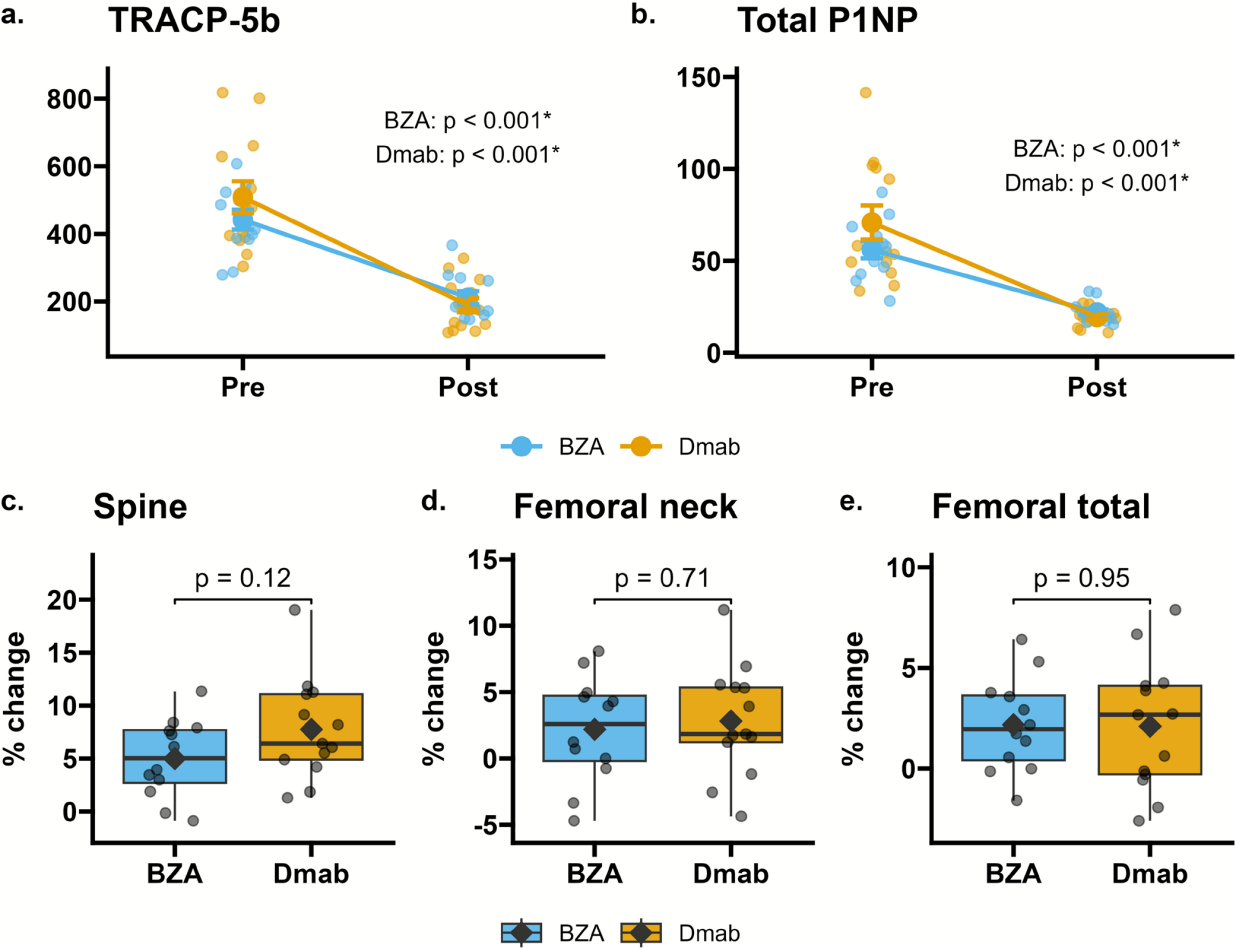
Bone turnover marker and percentage change in bone mineral density (BMD). (a) Change in TRACP‐5b (tartrate‐resistant acid phosphatase type 5b; reference range in women, 120–420 mU/dL). (b) Change in total P1NP (total N‐terminal propeptide of type 1 procollagen; reference range in postmenopausal women, 26.4–98.2 μg/L). Both BZA and Dmab significantly reduced marker levels (*p* < 0.001 for both). (c–e) Percentage change in BMD at (c) spine, (d) femoral neck, and (e) total femur; no significant group differences (all *p* > 0.05). Boxes show interquartile range with median; whiskers denote range.

After 12 months of treatment, TRACP‐5b and total P1NP significantly decreased in both groups (Figure 2a,b), indicating effective suppression of bone turnover. The mean increase in spine BMD was 5.0% in the BZA and 7.8% in the Dmab, without significant differences (*p* = 0.12; Figure 2c). Similarly, there were no significant differences in femoral neck BMD (2.2% vs. 2.8%; Figure 2d) and femoral total BMD (2.2% vs. 2.1%; Figure 2e) at the 12‐month time point.

### 3.3 FEA

**FIGURE 3:**
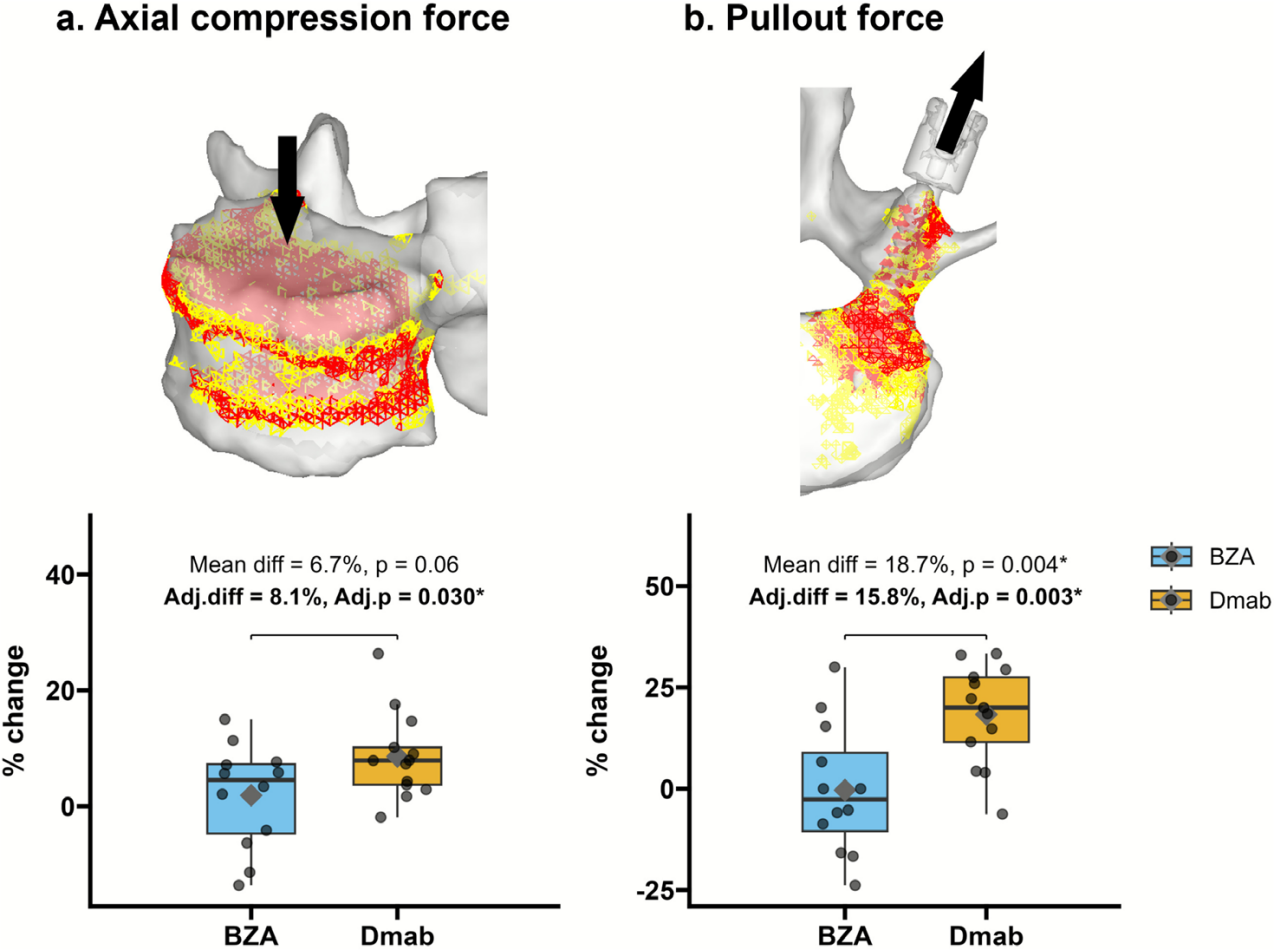
Vertebral compressive strength and screw pullout strength. (a) Percentage change from baseline in compressive strength in BZA versus Dmab groups; adjusted mean difference = 8.1%, *p* = 0.030. (b) Percentage change from baseline in pullout strength; adjusted mean difference = 15.8%, *p* = 0.003. Boxes show interquartile range with median; whiskers denote range. Adjusted (adj.) *p* values based on IPTW adjusted multivariable regression model. The above images show the distribution of elements associated with high compressive risk, yielding elements (yellow) and failure elements (red), for both compression and screw pullout strength.

**FIGURE 4:**
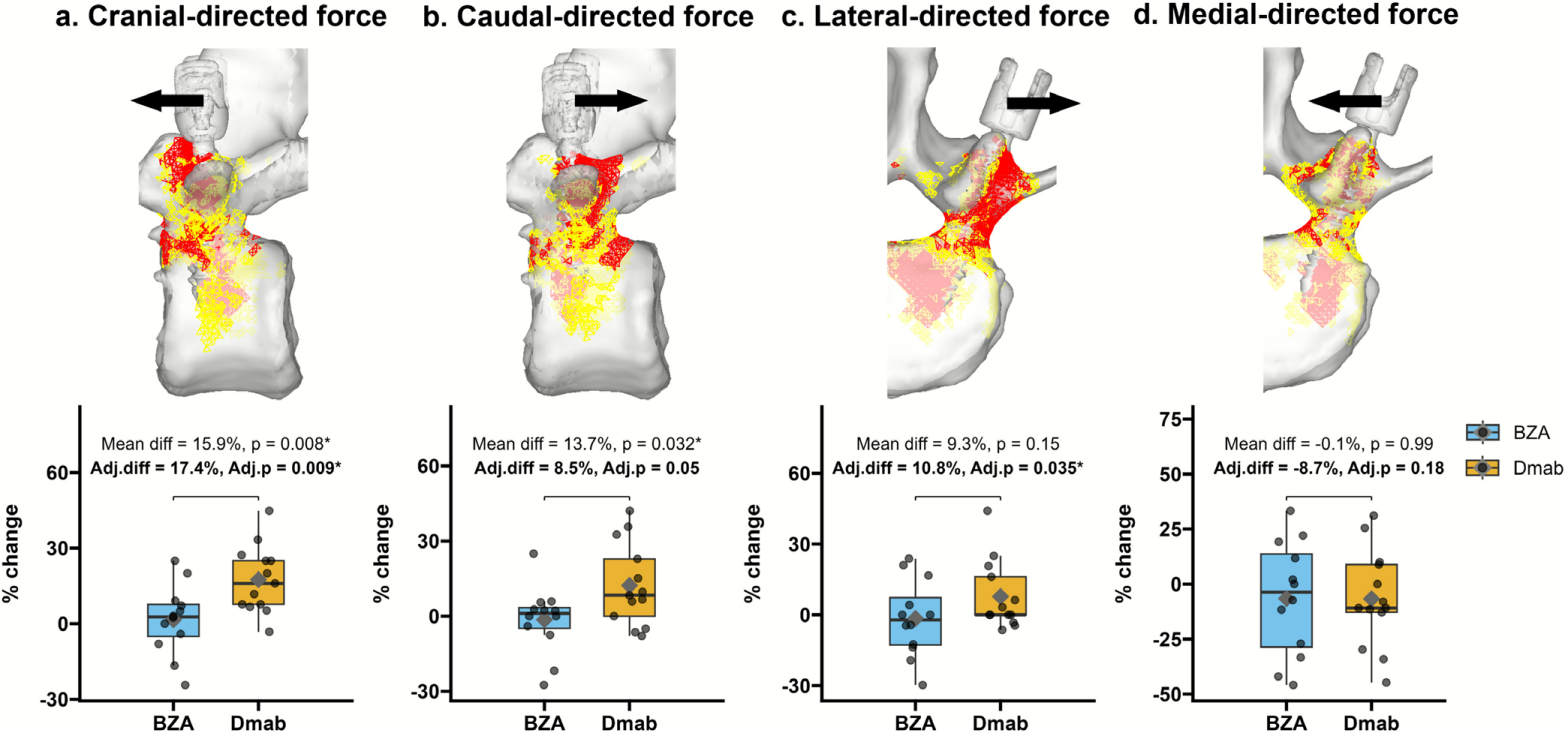
Multidirectional pedicle screw fixation strength. (a–d) Percentage change from baseline in multidirectional screw fixation strength, comparing BZA and Dmab. Cranial (adjusted mean difference: 17.4%, *p* = 0.009) and lateral (10.8%, *p* = 0.035) directions showed significantly greater strength in the Dmab group. Adjusted (adj.) *p* values based on IPTW adjusted multivariable regression model. Boxes show interquartile range with median; whiskers denote range. The above images show the distribution of elements associated with high compressive risk, yield elements (yellow) and failure elements (red), for both compression and screw pullout strength.

Representative stress and strain maps are shown in Figures 3a,b and 4a–d to visualize internal load distribution and failure elements, which were consistent with previous studies [26, 27, 28, 29]. The mean compression force and POS were 4657.0 ± 1266.7 and 430.4 ± 105.2 N at baseline in our cohort, respectively.

Univariate analysis showed an unadjusted mean difference of 6.7% (*p* = 0.06) between groups in vertebral compression force (Figure 3a). After IPTW in the multivariable regression, the Dmab group showed a significantly greater improvement compared to the BZA group, with an adjusted mean difference of 8.1% (95% CI, 0.9–15.3; *p* = 0.030). Other baseline variables had no significant effect on compression strength.

POS significantly increased in the Dmab group compared to the BZA group (unadjusted mean difference: 18.7%; *p* = 0.004), a finding consistent after IPTW adjustment in multivariable analysis (adjusted mean difference: 15.8% [95% CI, 6.2–25.4], *p* = 0.003; Figure 3b). Additionally, BMI was significantly associated with increased POS (β = 2.1 ± 0.9, *p* = 0.030). Pearson correlation analysis showed no significant correlation between POS and spine DXA change (correlation coefficient = 0.33, *p* = 0.11).

The percentage change in the cranial‐directed force was significantly greater in the Dmab group compared with the BZA group (adjusted mean difference: 17.4% [95% CI, 4.9–30.0], *p* = 0.009; Figure 4a). Although the caudal‐directed force showed a numerical increase in the Dmab group (adjusted mean difference: 8.5% [95% CI, −0.0–17.1], *p* = 0.051; Figure 4b), this difference did not reach statistical significance. The lateral‐directed force also showed a statistically significant difference favoring the Dmab group (adjusted mean difference: 10.8% [95% CI, 0.9–20.8], *p* = 0.035; Figure 4c). Conversely, medial‐directed force strength showed no significant treatment effect (adjusted mean difference: −8.8% [95% CI, −21.8–4.3], *p* = 0.18; Figure 4d). Additionally, BMI was positively associated with caudal‐directed force across all models (*β* = 2.2 ± 0.6, *p* = 0.002).

### 3.4 IPTW Diagnostics and Sensitivity Analysis

The propensity score distributions showed moderate overlap between groups, with 64% of observations lying within the common support (Figure S4a). IPTW weights yielded an effective sample size of 21.2 (Figure S4bF1). Absolute SMDs decreased after weighting for all covariates except spine DXA *T* score, suggesting that weighting effectively reduced confounding across most baseline variables (SMD = 0.32; Figure S4c).

Sensitivity analyses confirmed the robustness of the main findings. Significant associations between treatment and increases in compression force (adjusted mean difference: 8.3%; *p* = 0.045) and POS (16.8%; *p* = 0.009) were observed even without IPTW, consistent with the weighted analyses. Cranial‐directed force also showed consistent differences with (17.4%; *p* = 0.009) and without IPTW (16.4%; *p* = 0.028; Table S2). Stabilized IPTW models yielded comparable results for compression force, POS, and multidirectional loading parameters (Table S3).

## 4 Discussion

This study compared the effects of antiresorptive drugs, Dmab and BZA, on pedicle screw fixation using FEA in postmenopausal osteoporosis patients. Multivariable regression analyses performed 1 year after treatment initiation revealed significantly greater improvements with Dmab, especially in POS and cranial/lateral‐directed forces. Additionally, BMI was independently associated with POS and caudal‐directed force strength. To our knowledge, this study is the first to comparatively evaluate two types of antiresorptive osteoporosis agents on pedicle screw constructs using FEA, highlighting the potential advantages of Dmab in osteoporotic spinal surgery.

Our study confirmed a significantly greater improvement in compression strength with Dmab compared to BZA which aligns with the 8.1% increase in vertebral strength shown in the FREEDOM trial with FEA at 12 months [30]. While no prior FEA study specifically examined BZA's effect on vertebral bodies, clinical trials have reported a greater relative risk reduction in fractures with Dmab (70%) compared to BZA (40%) [15, 31]. Our FEA findings support these clinical efficacy differences from a biomechanical standpoint.

Optimizing bone health is essential for successful spinal instrumentation surgery. Our study revealed a significant 15.8% greater improvement in POS with Dmab compared to BZA, despite the absence of statistically significant differences in spine BMD changes as measured by DXA. While prior research has traditionally identified BMD as a key determinant of POS [32], more recent studies advocated for the utility of regional BMD, specifically focusing on the area surrounding screw placement [33]. Clinical data indicate that raloxifene increases BMD by 2%–3% at 12 months, while Dmab leads to a 5%–6% increase, based on findings from independent trials [31, 34]. Despite the small sample size, we observed a trend in BMD changes that aligned with these previous findings. Interestingly, the mild correlation observed between BMD and POS in the present study reflects the inherent limitations of DXA as a two‐dimensional method for assessing mechanical strength.

Pedicle screws are subjected to non‐axial three‐dimensional loads both intraoperatively and postoperatively [35, 36]. A cadaveric study indicated that laterally and cranially directed forces significantly contribute to bone deformation in the vertebral body [36]. Our study demonstrated a 17.4% increase in Dmab compared to BZA, suggesting a promising contribution of Dmab to enhanced screw stability against these critical loads. The changes in caudally directed force were limited (adjusted mean difference: 8.5%, *p* = 0.05), potentially due to structural differences in the vertebra around the screw tip. Cranially directed force, with the pedicle acting as a fulcrum, transmits force from the screw tip to the cancellous bone of the vertebral body in addition to the strain on the pedicle. In contrast, caudally directed force generates torque toward the cranial side around the screw tip, primarily loading the cortical endplate region. Although both Dmab and BZA improve bone microarchitecture, their effects on peri‐implant mechanical strength may differ [37]. These microarchitectural differences at the screw tip could also influence the assessment of laterally and medially directed forces. Lateral moment at the screw head generates a medially directed torque at the tip. The distinct difference in lateral force may reflect a mechanism similar to that underlying cranial‐caudal force variation, in which peri‐implant bone plays a key role. Given that trabecular bones are commonly involved from the early phases of primary osteoporosis, drug‐specific regional actions may lead to different biomechanical outcomes in spinal instrumentation across a broad patient population [37, 38, 39].

There are several limitations that need to be addressed in this study. First, the relatively small number of patients included may limit statistical power, although the sample size was determined pragmatically based on approximate estimates from prior FEA studies. As the effect size and variance used for power calculations were borrowed from a vehicle‐controlled study, mis‐specification in an active‐comparator setting is possible and may have led to under‐ or over‐powering. Second, treatment allocation was not randomized and relied on patient preference after a neutral explanation provided by treating physicians. Clinicians could still have influenced choices. In addition, factors such as aversion to injections, perceived convenience or prior medication experience may have affected treatment selection. Third, residual confounding, despite adjustment for spine BMD *T* score, might have influenced outcomes that were not fully accounted for in our analysis. Fourth, secondary osteoporosis was excluded based on FRAX‐guided interviews and laboratory assessments; however, some misclassification may remain without dedicated imaging confirmation. Fifth, the follow‐up period of 1 year may be insufficient to evaluate long‐term effects of antiresorptive treatments on pedicle screw fixation strength. Lastly, while FEA provides valuable biomechanical insights, the clinical implications of these biomechanical improvements require validation through longer‐term clinical outcomes studies. Direct experimental validation of drug effects on human vertebrae is not feasible for ethical and practical reasons. Nevertheless, CT‐based finite element methods similar to ours have been rigorously validated against cadaveric mechanical testing [40, 41], and have shown strong predictive value for vertebral fracture outcomes in large human cohorts [42, 43]. These prior validations support the reliability of this approach in estimating vertebral mechanical strength. Future randomized, controlled studies with larger sample sizes and extended follow‐up periods are warranted to confirm and expand upon these preliminary findings.

In conclusion, Dmab was associated with enhanced POS and resistance to cranial and lateral directional forces compared to BZA. These findings imply that Dmab may provide superior reinforcement of peri‐implant bone microarchitecture, thereby offering greater biomechanical support for pedicle screw stability. This study adds a layer to pharmacologic strategies aimed at optimizing bone health in osteoporotic patients undergoing spinal instrumentation.

## Author Contributions

T.A., S.T., and T.T. contributed to data analysis and curation. S.T. and Y.K. were involved in data collection. T.A. prepared the initial draft of the manuscript. M.I., P.V., P.G.P., and B.A.A. contributed to data interpretation and critical revision of the manuscript. K.I. conceived and designed the study, performed data analysis, wrote and revised the manuscript, supervised the study, and administered the project. All authors approved the final version of the manuscript.

## Funding

This work was supported by Japan Society for the Promotion of Science (16K10838, 19K18544, 22K12896, 22KK0265).

## Conflicts of Interest

P.P. reports research support from Globus, Medtronic, and Cerapedics, and receives royalties from Royal Biologics. K.I. reports consulting and research funding from Amgen Inc. None of these disclosure are related to this research work. The other authors declare no conflicts of interest.

## Supporting information


**Figure S1:** The mesh size assessment for the FEM model.
**Figure S2:** Finite element model of L4 vertebra and force–displacement curve.
**Figure S3:** Study diagram.
**Figure S4:** Inverse probability of treatment weights (IPTW) diagnostics.
**Table S1:** Material properties and modeling definitions used in the finite element analyses.
**Table S2:** Sensitivity analysis with and without IPTW in multivariable regression analyses.
**Table S3:** Sensitivity analysis with stabilized IPTW.

## Data Availability

The data that support the findings of this study are available in the Supporting Information of this article.

## References

[1] E. Rometsch , M. Spruit , J. E. Zigler , et al., “Screw‐Related Complications After Instrumentation of the Osteoporotic Spine: A Systematic Literature Review With Meta‐Analysis,” Global Spine Journal 10 (2020): 69–88, 10.1177/2192568218818164. 32002352 PMC6963360

[2] P. A. Anderson , N. C. Binkley , and J. T. Bernatz , “Bone Health Optimization (BHO) in Spine Surgery,” Spine 48 (2023): 782–790, 10.1097/brs.0000000000004618. 36917718

[3] K. Ishikawa , S. Tani , T. Toyone , et al., “The Potential Effect of Romosozumab on Perioperative Management for Instrumentation Surgery,” JOR Spine 7 (2024): e1356, 10.1002/jsp2.1356. 39104831 PMC11299907

[4] Y. A. Al‐Najjar , D. A. Quraishi , N. Kumar , and I. Hussain , “Bone Health Optimization in Adult Spinal Deformity Patients: A Narrative Review,” Journal of Clinical Medicine 13 (2024): 4891, 10.3390/jcm13164891. 39201032 PMC11355164

[5] S. L. Silverman , J. T. Schousboe , and D. T. Gold , “Oral Bisphosphonate Compliance and Persistence: A Matter of Choice?,” Osteoporosis International 22 (2011): 21–26, 10.1007/s00198-010-1274-6. 20458571 PMC3017316

[6] I. Imaz , P. Zegarra , J. Gonzalez‐Enriquez , B. Rubio , R. Alcazar , and J. M. Amate , “Poor Bisphosphonate Adherence for Treatment of Osteoporosis Increases Fracture Risk: Systematic Review and Meta‐Analysis,” Osteoporosis International 21 (2010): 1943–1951, 10.1007/s00198-009-1134-4. 19967338

[7] D. J. Torgerson and S. E. Bell‐Syer , “Hormone Replacement Therapy and Prevention of Nonvertebral Fractures: A Meta‐Analysis of Randomized Trials,” JAMA 285 (2001): 2891–2897, 10.1001/jama.285.22.2891. 11401611

[8] S. R. Cummings , J. San Martin , M. R. McClung , et al., “Denosumab for Prevention of Fractures in Postmenopausal Women With Osteoporosis,” New England Journal of Medicine 361 (2009): 756–765, 10.1056/NEJMoa0809493. 19671655

[9] I. R. Reid , P. D. Miller , J. P. Brown , et al., “Effects of Denosumab on Bone Histomorphometry: The FREEDOM and STAND Studies,” Journal of Bone and Mineral Research 25 (2010): 2256–2265, 10.1002/jbmr.149. 20533525

[10] C. Jeong , J. Ha , J. I. Yoo , et al., “Effects of Bazedoxifene/Vitamin D Combination Therapy on Serum Vitamin D Levels and Bone Turnover Markers in Postmenopausal Women With Osteopenia: A Randomized Controlled Trial,” Journal of Bone Metabolism 30 (2023): 189–199, 10.11005/jbm.2023.30.2.189. 37449351 PMC10345998

[11] I. Falsetti , G. Palmini , C. Aurilia , S. Donati , T. Iantomasi , and M. L. Brandi , “Selective Estrogen Receptor Modulators in Post‐Menopausal Osteoporosis,” International Journal of Bone Fragility 2 (2022): 93–96, 10.57582/ijbf.220203.093.

[12] S. Tani , K. Ishikawa , Y. Kudo , et al., “The Effect of Denosumab on Pedicle Screw Fixation: A Prospective 2‐Year Longitudinal Study Using Finite Element Analysis,” Journal of Orthopaedic Surgery and Research 16 (2021): 219, 10.1186/s13018-021-02360-2. 33771178 PMC7995779

[13] D. K. Chin , J. Y. Park , Y. S. Yoon , et al., “Prevalence of Osteoporosis in Patients Requiring Spine Surgery: Incidence and Significance of Osteoporosis in Spine Disease,” Osteoporosis International 18 (2007): 1219–1224, 10.1007/s00198-007-0370-8. 17387420

[14] D. C. Lee , A. Varela , P. J. Kostenuik , M. S. Ominsky , and T. M. Keaveny , “Finite Element Analysis of Denosumab Treatment Effects on Vertebral Strength in Ovariectomized Cynomolgus Monkeys,” Journal of Bone and Mineral Research 31 (2016): 1586–1595, 10.1002/jbmr.2830. 27149403

[15] S. L. Silverman , C. Christiansen , H. K. Genant , et al., “Efficacy of Bazedoxifene in Reducing New Vertebral Fracture Risk in Postmenopausal Women With Osteoporosis: Results From a 3‐Year, Randomized, Placebo‐, and Active‐Controlled Clinical Trial,” Journal of Bone and Mineral Research 23 (2009): 1923–1934, 10.1359/jbmr.080710. 18665787

[16] S. F. Anderson , “Best (but Oft Forgotten) Practices: Sample Size Planning for Powerful Studies,” American Journal of Clinical Nutrition 110 (2019): 280–295, 10.1093/ajcn/nqz058. 31131390

[17] A. G. O'Keeffe , G. Ambler , and J. A. Barber , “Sample Size Calculations Based on a Difference in Medians for Positively Skewed Outcomes in Health Care Studies,” BMC Medical Research Methodology 17 (2017): 157, 10.1186/s12874-017-0426-1. 29197347 PMC5712177

[18] K. Ishikawa , T. Nagai , K. Tsuchiya , et al., “High Bone Turnover Status as a Risk Factor in Symptomatic Hypocalcemia Following Denosumab Treatment in a Male Patient With Osteoporosis,” Clinical Interventions in Aging 13 (2018): 1929–1934, 10.2147/CIA.S180614. 30349211 PMC6183698

[19] K. Ishikawa , T. Nagai , K. Sakamoto , et al., “High Bone Turnover Elevates the Risk of Denosumab‐Induced Hypocalcemia in Women With Postmenopausal Osteoporosis,” Therapeutics and Clinical Risk Management 12 (2016): 1831–1840, 10.2147/TCRM.S123172. 27980413 PMC5147395

[20] K. Matsukawa , Y. Yanai , K. Fujiyoshi , T. Kato , and Y. Yato , “Depth of Vertebral Screw Insertion Using a Cortical Bone Trajectory Technique in Lumbar Spinal Fusion: Radiological Significance of a Long Cortical Bone Trajectory,” Journal of Neurosurgery. Spine 35 (2021): 601–606, 10.3171/2021.2.SPINE202229. 34388711

[21] K. Matsukawa , Y. Yato , H. Imabayashi , N. Hosogane , T. Asazuma , and K. Nemoto , “Biomechanical Evaluation of the Fixation Strength of Lumbar Pedicle Screws Using Cortical Bone Trajectory: A Finite Element Study,” Journal of Neurosurgery. Spine 23 (2015): 471–478, 10.3171/2015.1.Spine141103. 26161515

[22] Y. Matsuura , H. Giambini , Y. Ogawa , et al., “Specimen‐Specific Nonlinear Finite Element Modeling to Predict Vertebrae Fracture Loads After Vertebroplasty,” Spine 39 (2014): E1291–E1296, 10.1097/BRS.0000000000000540. 25077904 PMC4191996

[23] K. Imai , “Analysis of Vertebral Bone Strength, Fracture Pattern, and Fracture Location: A Validation Study Using a Computed Tomography‐Based Nonlinear Finite Element Analysis,” Aging and Disease 6 (2015): 180–187, 10.14336/AD.2014.0621. 26029476 PMC4441400

[24] J. H. Keyak , S. A. Rossi , K. A. Jones , and H. B. Skinner , “Prediction of Femoral Fracture Load Using Automated Finite Element Modeling,” Journal of Biomechanics 31, no. 2 (1998): 125–133, 10.1016/s0021-9290(97)00123-1. 9593205

[25] J. N. Weinstein , K. F. Spratt , D. Spengler , C. Brick , and S. Reid , “Spinal Pedicle Fixation: Reliability and Validity of Roentgenogram‐Based Assessment and Surgical Factors on Successful Screw Placement,” Spine 13 (1988): 1012–1018, 10.1097/00007632-198809000-00008. 3206294

[26] E. Baksiova , S. Ahuja , F. Arabatzi , and A. Tsouknidas , “Posterior Spinal Stabilization: A Biomechanical Comparison of Laminar Hook Fusion to a Pedicle Screw System,” Clinical Biomechanics 91 (2022): 105535, 10.1016/j.clinbiomech.2021.105535. 34837862

[27] Y. Chevalier , M. Matsuura , S. Kruger , et al., “The Effect of Cement Augmentation on Pedicle Screw Fixation Under Various Load Cases: Results From a Combined Experimental, Micro‐CT, and Micro‐Finite Element Analysis,” Bone & Joint Research 10 (2021): 797–806, 10.1302/2046-3758.1012.BJR-2020-0533.R1. 34894754 PMC8696523

[28] A. Polikeit , S. J. Ferguson , L. P. Nolte , and T. E. Orr , “Factors Influencing Stresses in the Lumbar Spine After the Insertion of Intervertebral Cages: Finite Element Analysis,” European Spine Journal 12 (2003): 413–420, 10.1007/s00586-002-0505-8. 12955610 PMC3467788

[29] J. P. Grant , T. R. Oxland , and M. F. Dvorak , “Mapping the Structural Properties of the Lumbosacral Vertebral Endplates,” Spine 26 (2001): 889–896, 10.1097/00007632-200104150-00012. 11317111

[30] P. Zysset , D. Pahr , K. Engelke , et al., “Comparison of Proximal Femur and Vertebral Body Strength Improvements in the FREEDOM Trial Using an Alternative Finite Element Methodology,” Bone 81 (2015): 122–130, 10.1016/j.bone.2015.06.025. 26141837

[31] H. G. Bone , R. B. Wagman , M. L. Brandi , et al., “10 Years of Denosumab Treatment in Postmenopausal Women With Osteoporosis: Results From the Phase 3 Randomised FREEDOM Trial and Open‐Label Extension,” Lancet Diabetes and Endocrinology 5 (2017): 513–523, 10.1016/S2213-8587(17)30138-9. 28546097

[32] S. D. Cook , S. L. Salkeld , T. Stanley , A. Faciane , and S. D. Miller , “Biomechanical Study of Pedicle Screw Fixation in Severely Osteoporotic Bone,” Spine Journal 4 (2004): 402–408, 10.1016/j.spinee.2003.11.010. 15246300

[33] K. Ishikawa , T. Toyone , T. Shirahata , et al., “A Novel Method for the Prediction of the Pedicle Screw Stability: Regional Bone Mineral Density Around the Screw,” Clinical Spine Surgery 31 (2018): E473–E480, 10.1097/bsd.0000000000000703. 30102636

[34] B. Ettinger , D. M. Black , B. H. Mitlak , et al., “Reduction of Vertebral Fracture Risk in Postmenopausal Women With Osteoporosis Treated With RaloxifeneResults From a 3‐Year Randomized Clinical Trial,” Journal of the American Medical Association 282 (1999): 637–645, 10.1001/jama.282.7.637. 10517716

[35] X. Wang , C. E. Aubin , H. Labelle , S. Parent , and D. Crandall , “Biomechanical Analysis of Corrective Forces in Spinal Instrumentation for Scoliosis Treatment,” Spine 37 (2012): E1479–E1487, 10.1097/BRS.0b013e3182706745. 23151872

[36] R. J. Bianco , C. E. Aubin , J. M. Mac‐Thiong , E. Wagnac , and P. J. Arnoux , “Pedicle Screw Fixation Under Nonaxial Loads: A Cadaveric Study,” Spine 41 (2016): E124–E130, 10.1097/BRS.0000000000001200. 26571161

[37] M. Saito , Y. Kida , T. Nishizawa , et al., “Effects of 18‐Month Treatment With Bazedoxifene on Enzymatic Immature and Mature Cross‐Links and Non‐Enzymatic Advanced Glycation End Products, Mineralization, and Trabecular Microarchitecture of Vertebra in Ovariectomized Monkeys,” Bone 81 (2015): 573–580, 10.1016/j.bone.2015.09.006. 26385255

[38] R. M. Zebaze , A. Ghasem‐Zadeh , A. Bohte , et al., “Intracortical Remodelling and Porosity in the Distal Radius and Post‐Mortem Femurs of Women: A Cross‐Sectional Study,” Lancet 375 (2010): 1729–1736, 10.1016/S0140-6736(10)60320-0. 20472174

[39] S. Gamsjaeger , W. Brozek , R. Recker , K. Klaushofer , and E. P. Paschalis , “Transmenopausal Changes in Trabecular Bone Quality,” Journal of Bone and Mineral Research 29 (2014): 608–617, 10.1002/jbmr.2073. 23966337

[40] R. P. Crawford , C. E. Cann , and T. M. Keaveny , “Finite Element Models Predict In Vitro Vertebral Body Compressive Strength Better Than Quantitative Computed Tomography,” Bone 33 (2003): 744–750, 10.1016/s8756-3282(03)00210-2. 14555280

[41] X. Wang , A. Sanyal , P. M. Cawthon , et al., “Prediction of New Clinical Vertebral Fractures in Elderly Men Using Finite Element Analysis of CT Scans,” Journal of Bone and Mineral Research 27 (2012): 808–816, 10.1002/jbmr.1539. 22190331 PMC3510751

[42] D. L. Kopperdahl , T. Aspelund , P. F. Hoffmann , et al., “Assessment of Incident Spine and Hip Fractures in Women and Men Using Finite Element Analysis of CT Scans,” Journal of Bone and Mineral Research 29 (2014): 570–580, 10.1002/jbmr.2069. 23956027 PMC3925753

[43] L. J. Melton, 3rd , B. L. Riggs , G. H. van Lenthe , et al., “Contribution of In Vivo Structural Measurements and Load/Strength Ratios to the Determination of Forearm Fracture Risk in Postmenopausal Women,” Journal of Bone and Mineral Research 22 (2007): 1442–1448, 10.1359/jbmr.070514. 17539738

