# Supplementary material for: Comparative Finite Element Analysis of Denosumab and Bazedoxifene on Pedicle Screw Stability in Osteoporotic Spines
Source: JOR Spine. 2025 Dec 9;8(4):e70147. doi: 10.1002/jsp2.70147 (PMC12687563; doi:10.1002/jsp2.70147)
Supplement: Supplementary file 1 — Figure S1: The mesh size assessment for the FEM model. Figure S2: Finite element model of L4 vertebra and force–displacement curve. Figure S3: Study diagram. Figure S4: Inverse probability of treatment weights (IPTW) diagnostics. Table S1: Material properties and modeling definitions used in the finite element analyses. Table S2: Sensitivity analysis with and without IPTW in multivariable regression analyses. Table S3: Sensitivity analysis with stabilized IPTW. [file JSP2-8-e70147-s001.docx]

**Supplemental data (Figure S1-4, Table S1-3)**

**Comparative Finite Element Analysis of Denosumab and Bazedoxifene on Pedicle Screw Stability in Osteoporotic Spines**

Tomoyuki Asada^1^, Soji Tani^2^, Tomoko Towatari^2^, Mahoko Ishikawa^3^, Philip Varnadore^3^, Yoshifumi Kudo^2^, Peter Passias^3^, Benjamin Alman^3^, Koji Ishikawa*^2, 3^

1. Hospital for Special Surgery, NY, USA
2. Department of Orthopedic Surgery, Showa Medical University, Tokyo, Japan
3. Department of Orthopaedic Surgery, Duke University, NC, USA

***Corresponding author**: Koji Ishikawa (K.I)

Department of Orthopaedics, Duke University, 308 Research Drive, LSRC B330A, Durham, NC 27710, USA

Department of Orthopaedic Surgery, School of Medicine, Showa University, Tokyo, Japan

Address: Building No.6, room517, 1-5-8 Hatanodai, Shinagawa, Tokyo 142-8666, Japan

Tel: +81 3 3784 8543 Fax: +81 3 3784 9005

Email: [koji.ishikawa@duke.edu](mailto:koji.ishikawa@duke.edu)

**Contributions**

T.A., S.T., and T.T. contributed to data analysis and curation. S.T. and Y.K. were involved in data collection. T.A. prepared the initial draft of the manuscript. M.I., P.V., P.P., and B.A. contributed to data interpretation and critical revision of the manuscript. K.I. conceived and designed the study, performed data analysis, wrote and revised the manuscript, supervised the study, and administered the project. All authors approved the final version of the manuscript.

ORCID: https://orcid.org/0000-0003-3689-5662

**Supplemental Figure 1. The mesh size assessment for the FEM model**

**
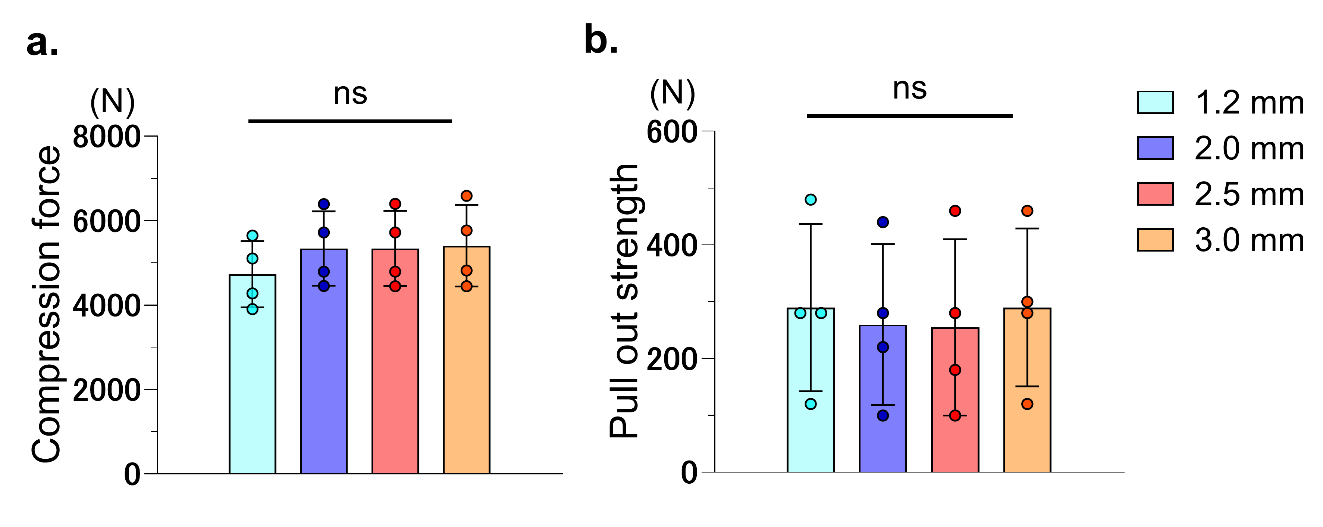
**

The mesh size assessment was performed with four types of sizes (1.2 mm, 2.0 mm, 2.5 mm, and 3.0 mm) across 4 patients. There were no differences in compression force (a) and pullout strength (b) among the sizes [Compression force (N, mean); 1.2 mm: 4732.7 / 2 mm: 5340.0 / 2.5 mm: 5340.5 / 3 mm: 5404.7, respectively], [Pull out strength (N, mean); 1.2 mm: 290.0 / 2 mm: 260.0 / 2.5 mm: 255.0 / 3 mm: 290.0, respectively]. Statistical analysis performed with One-way ANOVA.

**Supplemental Figure 2. Finite element model of L4 vertebra and force-displacement curve**

**
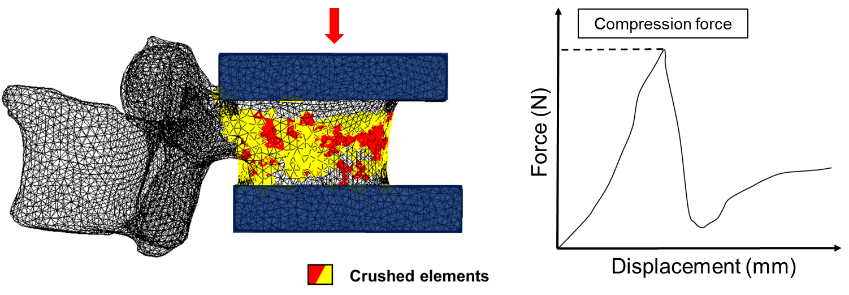
**

A finite element model of L4 vertebra with caps of poly(methyl-methacrylate) (PMMA) and a typical force-displacement curve illustrating compression force.

**Supplemental Figure 3. Study diagram**

**
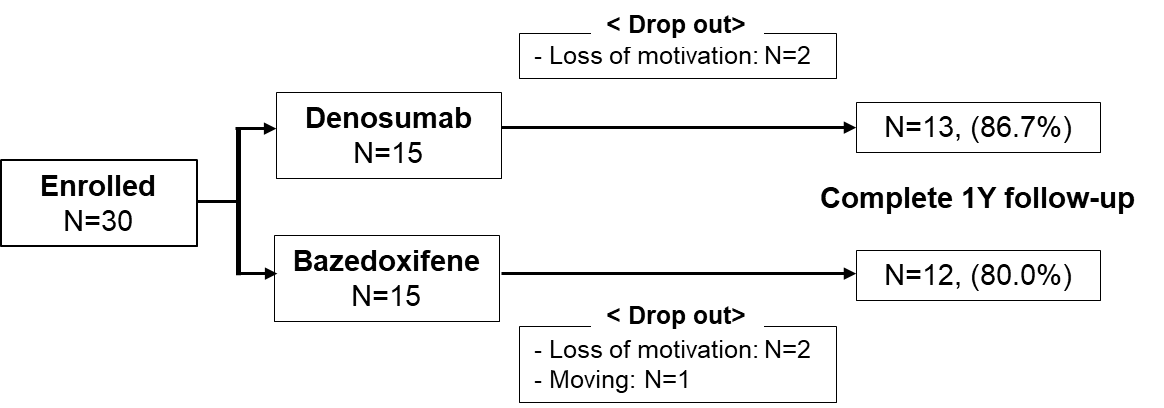
**

Patients were divided into two treatment groups: Denosumab (15 patients) and Bazedoxifene (15 patients). One-year follow-up was completed by 13 patients (86.7%) in the Denosumab group and 12 patients (80.0%) in the Bazedoxifene group.

**
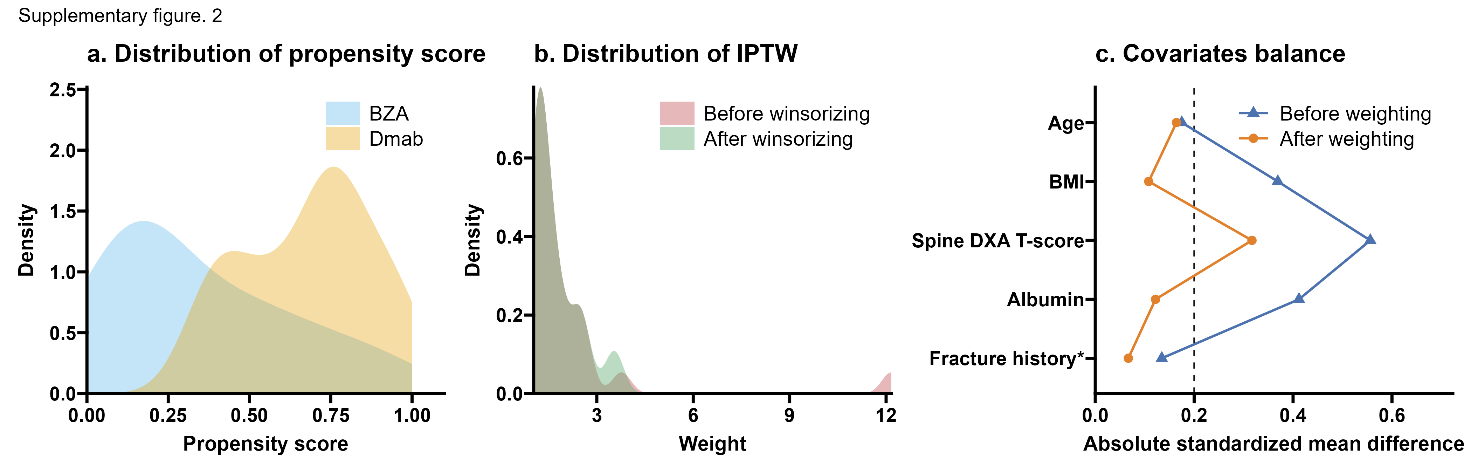
Supplemental Figure 4. Inverse Probability of Treatment Weights (IPTW) diagnostics**

1. Distribution of propensity scores for the bazedoxifene (BZA, light blue) and denosumab (Dmab, light orange) groups, showing moderate overlap between groups (BZA 0.039–0.918, Dmab 0.379–0.965; empirical common support 0.379–0.918), with 64% of observations lying within the common support (BZA 42%, Dmab 85%).
2. Distribution of IPTW before and after winsorizing at the 95th percentile, demonstrating the right-skewed weight distribution and capping of extreme values. It yielded an effective sample size of 21.2.
3. Standardized mean differences (SMDs) of baseline covariates before and after weighting. Covariate balance before weighting (age = 0.18, BMI = 0.37, spine DXA T-score = 0.56, Albumin = 0.41, Fracture history = 0.13) improved after weighting (age = 0.16, BMI = 0.11, spine DXA T-score = 0.32, Albumin = 0.12, Fracture history = 0.07), although a moderate imbalance remained for spine DXA T-score. Asterisk (*) indicates binary variable.

**Supplemental Table 1.** Material properties and modeling definitions used in the finite element analyses

| Component | Material / Region | Elastic Modulus | Poisson’s ratio | Yield Stress | Element Type | Mesh size/Thickness | Reference(s) |
| --- | --- | --- | --- | --- | --- | --- | --- |
| Vertebral bone | Patient-specific cancellous/cortical distribution (HU → vBMD) | vBMD via Keyak relationship ^a^ (elementwise) | 0.4 | Drucker–Prager equivalent stress ≥ element yield stress (Keyak ^b^) | Tetrahedral solid | 2.0 mm/ — | Keyak et al., 1998; Imai, 2015 |
| Cortical shell plates | Cortical bone shell representation | 10 GPa | 0.4 | Same as bone elements | Triangular plate | 2.0 mm/ 0.4mm | Imai, 2015; Tani, 2021 |
| Cement caps | PMMA | 2.5 GPa | 0.35 | 20.6 MPa | Tetrahedral solid | 2.0 mm/ — | Matsuura et al., 2014 |
| Pedicle screw | Titanium alloy (Ti–6Al–4V) | 110 GPa | 0.28 | 900 MPa | Tetrahedral solid | 1.0 mm/ — | Matsukawa et al., 2015; Manufacturer data |

FE, finite element; FEA, finite element analysis; HU, Hounsfield unit; vBMD, volumetric bone mineral density; PMMA, poly(methyl methacrylate); Ti–6Al–4V, titanium alloy; µε, microstrain.

1. Equation: $E= \left\{ \begin{aligned} 0.001 \left( \rho=0 \right) \\ 33900\rho^{2.20}\left( 0< \rho\leq0.27 \right) \\ 5307\rho+469\left( 0.28< \rho\leq0.6 \right) \\ 10200\rho^{2.01}(0.6\leq\rho) \end{aligned} \right\}$; b. $\sigma= \left\{ \begin{aligned} 1.0*{10}^{20} \left( \rho\leq0.2 \right) \\ 137\rho^{1.80}\left( 0.2< \rho\leq0.317 \right) \\ 114\rho^{1.72}(0.317<\rho) \end{aligned} \right\}$

**Supplemental Table 2. Sensitivity analysis with and without IPTW in multivariable regression analyses.**

|  | **Without IPTW** | | | | **With IPTW** | | | |
| --- | --- | --- | --- | --- | --- | --- | --- | --- |
|  | **Estimate** | **SE** | **95% CI** | **p-value** | **Estimate** | **SE** | **95% CI** | **p-value** |
| **Compression** | **8.3** | **3.9** | **0.2 – 16.4** | **0.045*** | **8.1** | **3.4** | **0.9 – 15.3** | **0.030*** |
| **Pullout strength** | **16.8** | **5.7** | **4.8 – 28.8** | **0.009*** | **15.8** | **4.6** | **6.2 – 25.4** | **0.003*** |
| **Cranial** | **16.4** | **6.9** | **1.9 – 30.8** | **0.028*** | **17.4** | **6.0** | **4.9 – 30.0** | **0.009*** |
| **Caudal** | 8.9 | 6.7 | -5.0 **–** 22.9 | 0.20 | 8.5 | 4.1 | -0.0 **–** 17.1 | 0.05 |
| **Lateral** | 9.7 | 7.4 | -5.8 **–** 25.3 | 0.21 | **10.8** | **4.8** | **0.9 – 20.8** | **0.035*** |
| **Medial** | -9.2 | 9.2 | -28.5 **–** 10.1 | 0.33 | -8.8 | 6.2 | -21.8 **–** 4.3 | 0.18 |

IPTW, inverse probability of treatment weight; SE, standard error; 95% CI, 95% confidence interval

*p < 0.05. Values meeting the significance threshold are marked with an asterisk.

**Supplemental Table 3. Sensitivity analysis with stabilized IPTW.**

|  | **Estimate** | **SE** | **95% CI** | **p-value** |
| --- | --- | --- | --- | --- |
| **Compression** | **8.1** | **3.4** | **0.9 – 15.2** | **0.030*** |
| **Pullout strength** | **15.8** | **4.6** | **6.2 – 25.5** | **0.003*** |
| **Cranial** | **17.5** | **6.0** | **5.0 – 30.0** | **0.008*** |
| **Caudal** | 8.5 | 4.1 | -0.1 – 17.1 | 0.05 |
| **Lateral** | **10.8** | **4.8** | **0.9 – 20.8** | **0.035*** |
| **Medial** | -8.7 | 6.2 | -21.7 – 4.4 | 0.18 |

SE, standard error; 95% CI, 95% confidence interval

*p < 0.05. Values meeting the significance threshold are marked with an asterisk.
